# *Cordyceps sinensis* promotes immune regulation and enhances bacteriostatic activity of PA-824 via IL-10 in *Mycobacterium tuberculosis* disease

**DOI:** 10.1590/1414-431X20176188

**Published:** 2017-08-07

**Authors:** D.G. Li, Z.X. Ren

**Affiliations:** Department of Respiratory Medicine, East Medical District of Linyi People's Hospital, Linyi, Shandong, China

**Keywords:** Mycobacterium tuberculosis, *Cordyceps sinensis* (Berk.) Sacc., PA-824, IFN-γ, IL-10

## Abstract

PA-824 is a novel bicyclic nitroimidazole anti-tuberculosis (TB) drug. *Cordyceps sinensis* (Berk.) Sacc. (CS) was proven to be a good immunomodulatory compound. This research aimed to investigate the effect of CS on PA-824 in *Mycobacterium tuberculosis* (*M.tb*) infected mice (female CBA/J mice, 6 to 8 weeks of age and 20±2 g of weight). Mice were randomly assigned to 4 groups: PA-824, CS, PA-824+CS, and control. To verify the effect of PA-824 and CS on *M.tb*, after drug administration, mice lungs were harvested and bacterial colony formations were measured. Cells were isolated from infected lungs and spleens to analyze the percentage of CD4^+^ T cells (CD11a positive). Lung cells were cultured to detect the secretion of interferon-γ (IFN-γ) and interleukin-10 (IL-10) by ELISA. IFN-γ and IL-10 double-positive CD4^+^ cells in peripheral blood were measured by flow cytometry. The expression levels of IL-2 and IL-10 in mice lungs were analyzed by real-time PCR and western blot. Results showed that PA-824 combined with CS led to the lowest lung colony-forming units (CFU) counts among treated groups. Furthermore, this beneficial outcome might be associated with the decreased CD11a on CD4^+^ cells in mice lungs and spleens. Moreover, the suppressed secretion of IFN-γ and IL-10, and IL-10 expressions, as well as the decreased IFN-γ and IL-10 double-positive CD4^+^ cells in blood, could also be associated with the positive effect. However, no significant effect on IL-2 production was found. The combination of PA-824 and CS had more effective bacteriostatic and immunomodulatory effects on *M.tb* infected mice than PA-824 alone. In conclusion, CS has the potential to be an effective adjuvant in TB treatment.

## Introduction

Tuberculosis (TB) is still a major global health problem even with the slightly decreasing incidence in recent years (1). Anti-TB therapy relies on combined effect of bactericidal and anti-inflammatory drugs that can effectively reduce drug-resistance. The crucial point of TB treatment regimen is security and practicability. Ideally, a novel drug should be able to interact with other drugs freely, and without competing with or causing resistance of current drugs, especially multidrug-resistance. The nitroimidazooxazine PA-824 represents a new class of anti-tubercular drugs (2). PA-824 is one of the novel bicyclic nitroimidazole drugs for TB treatment that has already been in phase II clinical trials, and the other one is OPC-67683 (3). PA-824 has potent activity against *Mycobacterium tuberculosis* (*M.tb*) *in vitro*, and does not demonstrate cross-resistance to a variety of commonly used anti-TB drugs (4,5). Although antibacterial activity of PA-824 to *M.tb* and multidrug resistance were evaluated, the exact mechanism is not well known (6,7).

*Cordyceps sinensis* (Berk.) Sacc. (CS) is a Chinese herbal. As an insect parasitizing fungus, it belongs to the ascomycete family, and is found at high altitudes in the Qinghai-Tibetan plateau. CS has a long reputation for being one of the most expensive raw materials used in Oriental Medicine (8). CS has a number of far reaching medicinal effects that have been proven by modern technical methods. For example, some water extracts of CS might be beneficial in the prevention of tumor metastasis (9). CS has already been used in respiratory ailment treatments, such as cough and phlegm, shortness of breath, bronchial discomfort, chronic obstructive pulmonary disease, and asthma (10,11). It also has been demonstrated that CS inhibits inflammatory reaction and prevented ischemic injury of many organs (12). However, the understanding of CS efficacy remains incomplete, as modern science attempts to investigate its effects in traditional medicine.

This study aimed to verify the antibacterial and immune regulation activities of CS on PA-824 application in *M.tb* infected mice.

## Material and Methods

### Preparation of PA-824 solution and CS extraction

The pure powder of PA-824 was provided by the Global Alliance for TB Drug Development through Research Triangle International (RTI Park, USA). For administration in mice, PA-824 was suspended in a cyclodextrin micelle formulation (CM-2) containing 10% hydroxypropyl-β-cyclodextrin (Sigma, USA) and 10% lecithin (ICN Pharmaceuticals Inc., USA) as previously described (13), and the suspensions were stored at 4°C. Aliquots were diluted in distilled water to the desired concentrations for dosing suspensions, and samples were shaken to ensure uniform dosing for oral administration.

CS produced in Qinghai, China, was purchased from the local wholesale distributor (Qinghai Cordyceps sinensis Technology Development, China). To obtain the extract of CS, 50 g of CS was dissolved in 100 mL distilled water. The CS solution was heated at 90°C for 2 h and concentrated by rotary evaporator (Eyela, Japan). Microfiltration was performed to remove bacteria and samples were lyophilized for 24 h. The CS extractions, weighting 13.6 g (27.2%) were diluted by adding distilled water to the concentrations that needed for esophageal gavage (14).

### Mice

All *in vivo* experiments were performed in pathogen-free female CBA/J mice (National Cancer Institute, USA), 6 to 8 weeks of age and 20±2 g of weight. The mice were maintained under level 3 biohazard conditions, provided with sterile chow and water *ad libitum*, and housed in constant temperature and humidity with 12-h light-dark cycling. The pathogen-free nature of the mouse was demonstrated by testing sentinel animals. All experimental protocols were approved by local Animal Care and Use Committee.

### Bacterial infections

*M.tb* strain Erdman (TMCC 107) was grown from low-passage seed lots in Proskauer-Beck liquid media (Seebio Biotech, China) containing 0.02% Tween 80 (Sigma) to mid-log phase, then frozen at –70°C until use. The CAB/J mice were infected with *M.tb* via the aerosol route by using the Inhalation Exposure System (Glas-Col, Inc., USA), with 5 mL of distilled water containing a suspension of bacteria that delivered about 100 bacteria/lung as previously described (15).

### PA-824 and CS intervention

Infected mice were randomly divided into four groups: control (no treatment), PA-824 (10 mg/kg), CS (200 mg/kg) and PA-824+CS (combination of 10 mg/kg PA-824 and 200 mg/kg CS). Treatment with PA-824 and/or CS started at the 20th day after infection, and was administered by esophageal gavage once daily (7 days/week for 8 weeks). Five additional mice were sacrificed before treatment to determine the bacterial load in the lungs. Quantitative cultures were performed by plating serial dilutions of individual partial organ homogenates onto nutrient Middlebrook 7H11 agar (Sigma-Aldrich, USA) with selective antibiotics as previously described (16,17), and bacterial colony formations were counted at the end of treatment. Data of lung bacterial colony-forming units (CFU) are reported as the log_10_ value of the mean number of bacteria recovered from four individual mice.

### Cells isolation from infected lung and spleen

Mice were euthanized by CO_2_ asphyxiation and the pulmonary cavities were opened. The lungs were then cleared of blood by pulmonary artery perfusion with 10 mL of saline containing 50 U/mL of heparin (Sigma). Then the lungs were harvested and placed in cold DMEM (Gibco, USA). After the connective tissue and trachea were removed, the lungs were disrupted by using sterile razor blades, and incubated for 30 min at 37°C in DMEM medium. Single cell suspensions were obtained from the lung tissue by using collagenase/DNase as previously described (18). Spleens were also harvested from mice and the cells dispersed via a nylon screen. Red blood cells were lysed using ammonium-chloride-potassium (ACK) lysis buffer (Sigma) and spleen cells were re-suspended in DMEM plus supplements (Gibco).

### Flow cytometry

Isolated cells for flow cytometry tests were obtained from lung or spleen and incubated with specific RPMI (Irvine Scientific, USA) supplemented with 0.1% sodium azide (Sigma-Aldrich) as previously described (18). Specific antibodies were purchased from BD Biosciences (PharMingen, USA): fluorescein isothiocyanate (FITC) labeled anti-CD11a (clone 2D7), and peridinin chlorophyll-protein labeled anti-CD4 (clone RM4-5). Appropriate isotype control antibodies (Ag, 25 μg/mL) were included in each analysis. Cells were analyzed using a FACS Calibur (BD Biosciences, USA) and data were analyzed using Cell Quest software (Becton Dickinson, USA).

### Lung cells culture

Lung cells were suspended at 5×10^6^ cells/mL in DMEM plus supplements (Gibco) and cultured with ovalbumin (OVA, 10 μg/mL, Sigma-Aldrich) or culture filtrate proteins from *M.tb* culture (10 μg/mL) for 5 days at 37°C with 5% CO_2_.

### Cytokine ELISA analysis

Supernatants of lung cell culture were harvested and the presence of interferon-γ (IFN-γ) and IL-10 were measured by ELISA. The primary antibodies IFN-γ (clone R4-6A2) and IL-10 (clone JES5.2A5) from BD PharMingen were used. The samples were dispensed in duplicate into the wells and standard curve was prepared using IFN-γ or IL-10 for each individual plate. Cytokine production was detected by the addition of a secondary biotinylated antibodies (IFN-γ, clone XMG1.2; IL-10, clone SXC-1; BD PharMingen) and followed by avidin-peroxidase and 3,3′,5,5′-tetramethylbenzidine (TMB) substrate system (Sigma).

### IFN-γ and IL-10 double-positive T cell detection

For cellular characterization of T cells in mice after bacterial infection and drug administration, the following murine anti-human monoclonal antibodies of IFN-γ and IL-10 (Becton Dickinson, Belgium) were used to direct immunofluorescence staining. In briefly, peripheral blood mononuclear cells from heparinized blood were obtained on a density gradient by Lymphoprep (Nicomed Pharma AS, Norway) as previously described (19). Negative controls included un-stimulated cells. The cells were suspended in RPMI 1640 and incubated for 16 h at 37°C. Then, cells were stained for membrane marker CD4^+^ T cells for 30 min at 4°C, followed by fixation for 10 min at room temperature using lyse/fix solution (Becton Dickinson). Next, permeabilization was performed using Perm 2 solution (Becton Dickinson) for 10 min at room temperature. After staining with anti-IFN-γ and anti-IL-10 antibodies for 1 h at 4°C, flow cytometric analysis was performed (20).

### Real-time PCR

Right medial lung lobes were homogenized in 1 mL of Ultraspec (Biotecx Laboratories, USA) and frozen rapidly at −80°C. Total cellular RNA was extracted from the homogenate and reverse transcribed by using the Omniscript RT kit (Qiagen, Germany) following the manufacturer’s instructions. Real-time PCR was performed using an iQ5 real-time PCR detection system (Bio-Rad, USA) using Taq-Man gene expression assays for Interleukin-2 (IL-2) and IL-10. The 2^-ΔΔCt^ method was used for relative quantification of mRNA expression and GAPDH was used as internal control (21).

### Western blot

The total proteins of right medial lung lobes were extracted according to the manufacturer’s protocol and the samples were detected using Pierce™ BCA Protein Assay Kit (Thermo Fisher Scientific, USA). Equal amounts of proteins (100 μg) were separated by sulfate-polyacrylamide gel electrophoresis and transferred onto nitrocellulose membranes. Western blotting was performed by standard techniques as described previously (22). The primary antibodies (1:1000) against IL-2 (D7A5), IL-10 (D13A11) and GAPDH (14C10) (Cell Signaling Technology, USA) were used followed with second antibody HRP goat anti-rabbit (1:1000, Cell Signaling Technology). All results are reported as changes of fluorescent band intensity of target antibody to GAPDH, which was used as an internal control. All sample bands intensity quantification was performed by using ImageJ software (National Institutes of Health, USA).

### Statistical analyses

Statistical analyses were performed by using Graphpad Prism 5 (Graphpad Software, USA). Results are reported as means±SD. Student's *t*-test was used for pairwise comparisons, a one-way analysis of variance (ANOVA) was used for multi-group comparisons. A P value of <0.05 was considered to be statistically significant.

## Results

### CFU was decreased in mouse lungs after administration of PA-824 and/or CS

**Figure 1.**
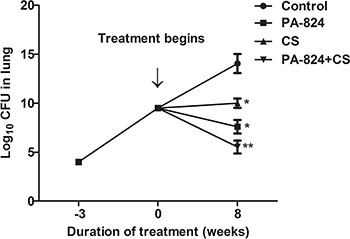
Quantitative analysis of colony-forming units (CFU) in mice lungs. Mice were infected with *Mycobacterium tuberculosis* Erdman strain for 3 weeks followed by 8 weeks of administration of PA-824, CS, or the combination of both. The bacterial CFU were decreased compared to the control without drug treatment. Data are reported as means±SD. CS: *Cordyceps sinensis* (Berk.) Sacc. *P*<*0.05, **P*<*0.01, compared to control (ANOVA).

As shown in Figure 1, after infection, lung bacterial CFU began to rise. Three weeks later, after PA-824 and/or CS treatment, the CFU counts were different among the 4 groups. After 8 weeks, lung CFU counts in mice lungs treated with PA-824 alone, CS alone and PA-824+CS were significantly decreased compared to the control group (P<0.05 or P<0.01). More important, the PA-824+CS group showed the lowest CFU counts, which could be indicative of an enhanced effect of CS on bactericidal activity of PA-824.

### Expressions of CD11a on CD4+ cells in lung and spleen were suppressed following PA-824 and/or CS treatment

**Figure 2.**
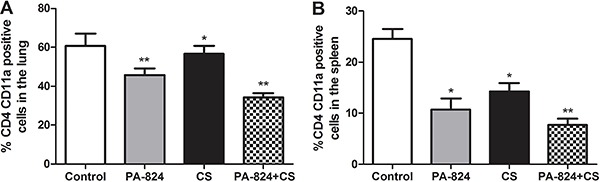
Expression of CD11a on CD4^+^ T cells isolated from the lung and spleen after treatment with PA-824, CS or the combination of both. Lung cells (*A*) and spleen cells (*B*) were isolated from *Mycobacterium tuberculosis*-infected mice throughout the course of the treatment and labeled with fluorescent antibodies specific for CD4 and CD11a. Data are reported as means±SD percentage of CD4 positive cells that expressed a high level of CD11a on their surface. CS: *Cordyceps sinensis* (Berk.) Sacc. *P*<*0.05, **P*<*0.01, compared to control (ANOVA).

To identify the mechanism by which treated mice could reduce the bacterial load in the lungs, we analyzed the level of T lymphocyte subset CD4 that entered the lung and spleen after 8 weeks of treatment. Cell adhesion molecules have been shown to be involved in cell recognition, signaling and autoimmune diseases. In this study, CD4^+^ T lymphocytes in lung were analyzed by measuring cell adhesion molecule CD11a expression. The group treated with PA-824 alone showed decreased CD11a expressions on CD4^+^ cells in lung compared with the control group. Meanwhile, in the PA-824+CS group, the CD11a expressions on CD4^+^ cells in lung were lower compared with the other groups (P<0.01 or P<0.05, Figure 2A). Analysis of the expressions of CD11a on CD4^+^ cells in the spleen of treated mice showed the similar result (P<0.05 or P<0.01, Figure 2B). These results suggested that PA-824 treatment decreased CD11a expressions on CD4^+^ T cells in lung and spleen and CS enhanced the efficacy of PA-824.

### IL-10 and IFN-γ productions were altered by PA-824 and CS combined effect

**Figure 3.**
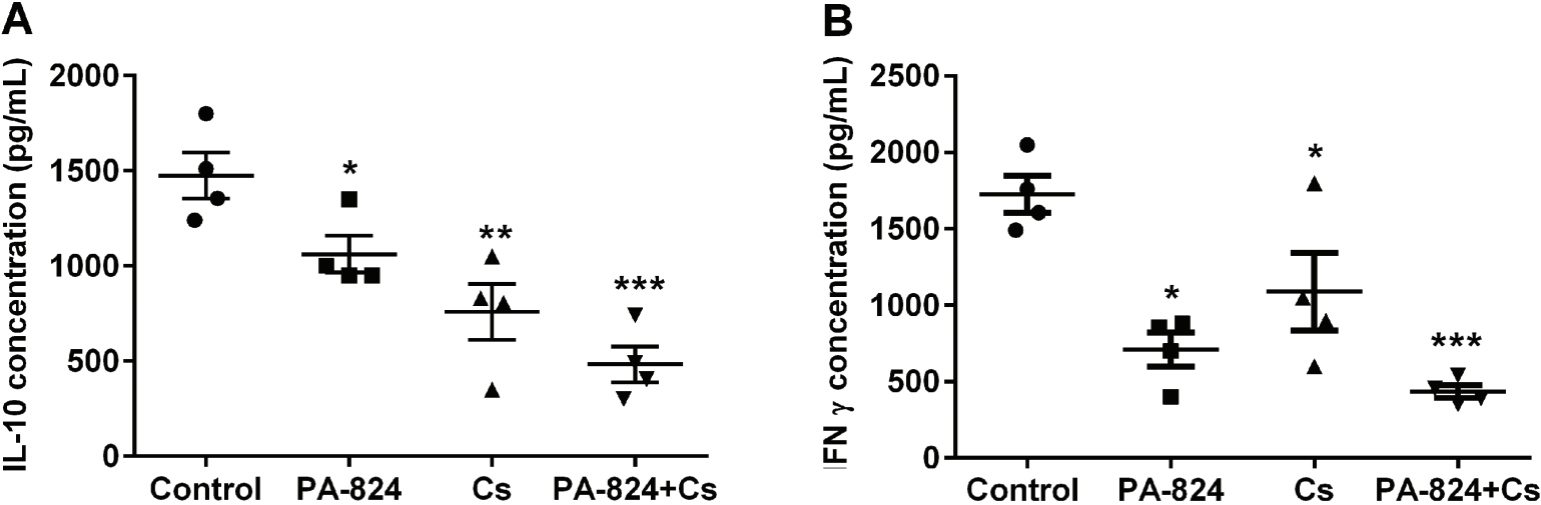
IL-10 and IFN-γ secretions in the infected mice lungs after administration of PA-824, CS or the combination of both. Lung cells from individual mice were cultured and IL-10 (*A*) and IFN-γ (*B*) secretions in the cell culture supernatants were measured by ELISA. Data are reported as means±SD. CS: *Cordyceps sinensis* (Berk.) Sacc.; IFN-γ: interferon-γ; IL-10: interleukin-10. *P*<*0.05, **P*<*0.01; ***P*<*0.001, compared to control (ANOVA).

**Figure 4.**
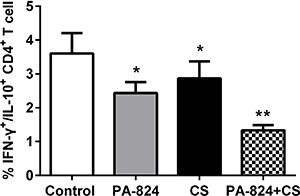
Relative IFN-γ and IL-10 double-positive CD4^+^ cells in peripheral blood of infected mice after administration of PA-824, CS or the combination of both, measured by flow cytometry. CS: *Cordyceps sinensis* (Berk.) Sacc.; IFN-γ: interferon-γ; IL-10: interleukin-10. *P*<*0.05, **P*<*0.01, compared to control (ANOVA).

To evaluate the specific responses of lymphocytes in the lungs of mice following *M.tb* infection and after drug treatments, IFN-γ and IL-10 productions in cultured lung cells were determined by ELISA. The results showed that IFN-γ and IL-10 were significantly lower in both PA-824- and CS-treated groups compared with the untreated control group (P<0.05 or P<0.01), while in the PA-824+CS group, the content of IFN-γ and IL-10 was reduced even more significantly (P<0.001) (Figure 3A and B). Similar results were found in cellular immunologic response detection as shown in Figure 4. The percentage of CD4^+^ T cells (IFN-γ and IL-10 double positive) in blood was decreased after PA-824 or CS administration compared with the untreated control group (P<0.05, or P<0.01). Moreover, in the PA-824+CS group, the number of IFN-γ and IL-10 double positive-CD4^+^ cells was the lowest of the 4 groups. These results suggested that CS could enhance the anti-inflammation effect of PA-824 *in vitro* and *in vivo*.

### Decreased IL-2 and IL-10 expressions in infected mice lungs after treatment with PA-824 and CS

**Figure 5.**
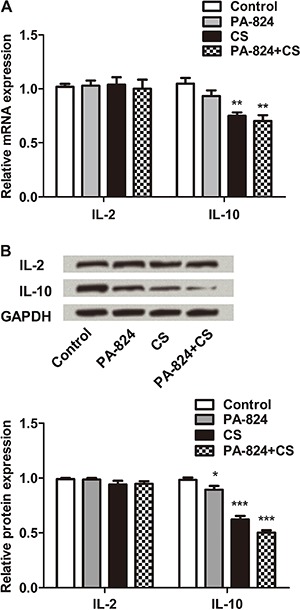
IL-2 and IL-10 expression in the lung during infection after administration of PA-824, CS or the combination of both. The mice lungs after treatment were stripped out and prepared for analysis. *A*, mRNA expression level of IL-2 and IL-10 in the mice lung by real-time PCR. *B*, Protein immunoblots and quantification of IL-2 and IL-10 in *M.tb* infected mice lungs after treatment. GAPDH acted as an internal control. CS: *Cordyceps sinensis* (Berk.) Sacc.; *M.tb*: *Mycobacterium tuberculosis*; IL-2: interleukin-2; IL-10: interleukin-10. *P*<*0.05, **P*<*0.01, ***P*<*0.001, compared to control (ANOVA).

To quantify cytokine production in the lung cells after treatment, cytokine expression levels in lung homogenates were measured. RT-PCR results are shown in Figure 5A; mRNA expression levels of IL-10 in CS and PA-824+CS groups were significantly decreased compared with control (P<0.01). The level was lower in the PA-824+CS group. However, no significant impact on IL-2 mRNA expression was found in the present experiment. Figure 5B shows the results of protein immunoblot and quantification for IL-2 and IL-10 by western blot assay. Consistent with the result of RT-PCR, PA-824 and CS reduced the IL-10 protein expression level (P<0.05 or P<0.001), while no significant effects were found on IL-2. Even more important, protein expression level of IL-10 was the lowest in the PA-824+CS group, suggesting that CS might enhance the efficacy of PA-824 via inhibition of IL-10 expression.

## Discussion

Almost all anti-TB drugs used in regular clinics were developed more than 50 years ago. There is limited research assessing the potential of traditional Chinese medicine as TB drug adjuvants in murine models, which might provide the theoretical basis for their use in clinical treatment (23). Meanwhile, the wide usage of antibiotics has led to the widespread emergence of resistant bacteria, which is also one of the very important reasons for *M.tb* multiple drug resistance (24). The treatment of drug-resistant TB by second-line drugs is an important component of TB control, as well as an integral part of the World Health Organization Stop TB Strategy (25). In order to effectively prevent and treat TB, anti-TB drugs with new structures or mechanisms are urgently needed. In this study, our results suggested that the antibacterial effect of PA-82 was enhanced by CS in *M.tb* infected murine model; we also present the possible mechanism.

PA-824 has a unique mechanism of action and has no cross-resistance to other existing TB drugs (4). In murine model, PA-824 was proved to have bactericidal activity during the initial and continuation phases of TB treatment (26). PA-824 also showed activity against latent or persistent *M.tb* isolates *in vivo* (27). In the present study, our results suggested that the addition of CS to PA-824 caused a more effective reduction of *M.tb* colony formation in lungs than PA-824 alone. We also found that CD11a expression on CD4^+^ cells was decreased after treatment with PA-824 and/or CS. CD11a, a cell adhesion molecule involved in cellular adhesion and costimulatory signaling, and that combines with CD18 to form the integrin lymphocyte function-associated antigen-1 (LFA-1) was expressed on all leukocytes. LFA-1 plays a central role in leukocyte intercellular adhesion, trafficking and activation through interactions with its ligands ICAMs 1-3 (intercellular adhesion molecules 1-3), and also functions in lymphocyte costimulatory signaling and thus it emerged as an attractive therapeutic target for treatment of multiple diseases (28). The decreased CD11a suggested that the inhibition effect of PA-824+CS enhanced the influence of PA-824 on TB infected mice lymphocyte.

CS has a variety of pharmacological effects, such as anti-inflammatory action, anti-apoptotic effect, stimulation of natural killer cells, and antitumor activity (12,29–31). CS anti-TB capsule combined with chemotherapy promoted a focused absorption, increased the sputum conversion rate, improved symptoms and enhanced the immunity of TB patients, suggesting better therapeutic effect compared with modern clinical drugs (32). IFN-γ is a cytokine with strong resistance to pathogenic microorganisms. IFN- γ is secreted by innate immune cells such as dendritic cells, antigen-presenting cells, and adaptive immune cells such as CD4^+^ T cell and CD8^+^ T cells. IL-10 is a multifunctional negative regulatory cytokine. Our results suggested that contents of IFN-γ and IL-10 in the supernatant of lung cell culture were decreased after drugs treatments. CS administration showed a more significant reduction of IFN-γ and IL-10 contents. It indicated that CS enhanced the action of PA-824 in murine lung cellular immune response.

As previously reported, IFN-γ^+^ and IL-10^+^ double-positive cells regulate immune responses to certain infections. For example, patients with either acute pulmonary tuberculosis, *Borrelia burgdorferi* or *Leishmania visceralis* infection expressed pathogen-specific IL-10^+^ and IFN-γ^+^ double-positive IFN-γ-secreting T helper 1 (Th1) cells in the blood and lung (33). In this study, our results suggested that IL-10^+^ and IFN-γ^+^ double-positive CD4^+^ T cells were decreased after PA-824 administration, and CS further enhanced PA-824 effect, suggesting that CS enhanced the regulation effect of PA-824 on cell-mediated immune response in TB injected mice.

The results showed that the expression of IL-10 in mice lung was significantly decreased, while there was no significant impact on IL-2. IL-10 is a potent immunomodulatory cytokine that directly or indirectly affects multiple cells *in vitro* (34). IL-10 is produced by a range of CD4^+^ T cell subsets, as well as by macrophages, dendritic cells, eosinophils B cells and mast cells; the major source of IL-10 is CD4^+^ Treg cells (33). IL-10 has broad anti-inflammatory properties, one of which is to counteract the function of Th1 lymphocytes. It has the dominant function to deactivate macrophages, resulting in diminished Th1 cytokine production, which might have far-reaching consequences on both innate and acquired immunity *in vivo* (21). In this regard, the immunosuppressive activity of IL-10 might contribute to mycobacterial disease (35). Research suggests that pathogens might cause high IL-10 expression to better sustain the infection process (36,37). Meanwhile, it has been suggested that removal of IL-10 enhanced protective immunity (38). Consistent with these studies, our results showed that PA-824 and CS suppressed the expression level of IL-10 in *M.tb* infected mice, and more importantly, the combination of PA-824 and CS significantly deepened this inhibition. These results imply that the effect of PA-824 on cellular immunologic response that was enhanced by CS might be related with the suppressed expression of IL-10.

In this study, the main finding was the enhanced activity of PA-824 by CS. As a traditional Chinese herbal, CS proved to be effective in promoting the PA-824 efficacy in the treatment of *M.tb* disease. In the development of anti-TB drugs, a major priority should be the ability to shorten the duration of TB treatment. Our results might also provide new perspectives for the use of traditional Chinese medicines combined with modern clinical drugs during clinical therapy, which might shorten the duration of tuberculosis treatment.
